# Association Between the Angioedema Control Test and Attack Frequency in Hereditary Angioedema

**DOI:** 10.1002/clt2.70143

**Published:** 2026-01-12

**Authors:** Aaron Yarlas, Alexandra J. Feld, Jakob B. Bjorner, Cary Thurm, Laura Bordone, Kenneth B. Newman, Sabrina Treadwell, Danny M. Cohn

**Affiliations:** ^1^ Ionis Pharmaceuticals Inc. Carlsbad California USA; ^2^ IQVIA Durham North Carolina USA; ^3^ Department of Vascular Medicine, Amsterdam Cardiovascular Sciences Amsterdam UMC University of Amsterdam Amsterdam the Netherlands

**Keywords:** AECT, disease control, HAE, patient‐reported outcome, validity

## Abstract

**Background:**

Hereditary angioedema (HAE), defined by unpredictable, painful, acute swelling attacks affecting several bodily locations, diminishes patients' quality of life. Assessing disease activity, burden, and treatment response pose challenges in routine care. The patient‐reported Angioedema Control Test (AECT) is a subjective measure of HAE disease control. Criterion validity of AECT with objective measures of disease control has not been previously assessed. This study evaluates the criterion validity of AECT using investigator‐confirmed HAE attack rate in patients with HAE.

**Methods:**

The Phase 3 OASIS‐HAE study (NCT05139810) randomized patients with HAE to receive donidalorsen 80 mg or placebo subcutaneously for 24 weeks. The full analysis population included all dosed patients (*N* = 90), pooled across treatment arms. This post‐hoc analysis examined the correlation between AECT and HAE attacks at Baseline, 12, and 24 weeks.

**Results:**

AECT scores correlated moderately to strongly with HAE attack rate (*ρ* = −0.40 to −0.85). Mean attack rates differed significantly between poor (AECT < 10) and well‐controlled (AECT ≥ 10) disease subgroups at study visits. At Week 24, 97.4% of patients reporting complete disease control (AECT maximum score of 16) had an attack rate of 0; the remaining patient had 1 attack. Patients with no attacks had a mean AECT score of 15.1 versus 7.7 for patients with attack rates > 0.

**Conclusion:**

This study supports the criterion validity of AECT in patients with HAE scores. AECT scores were strongly associated with objective disease control. AECT may be a valuable tool for monitoring disease control in patients with HAE.

## Graphical abstract

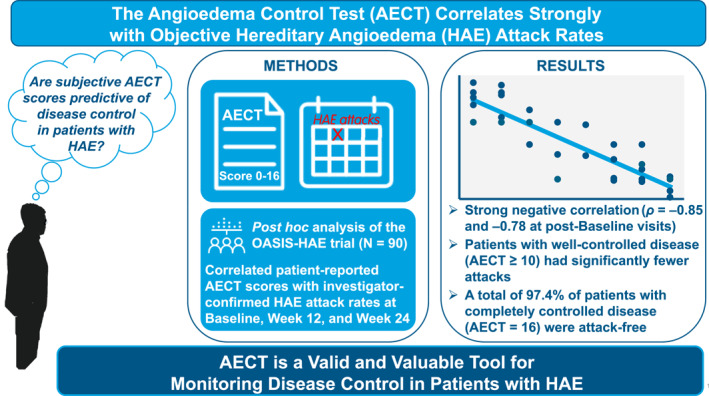

## 1 Introduction

Hereditary angioedema (HAE) is a rare, debilitating disease caused by increased bradykinin levels resulting in unpredictable recurrent episodes of severe swelling of the skin, gastrointestinal tract, upper respiratory system, face, and larynx, colloquially referred to as “HAE attacks” [1, 2, 3]. The true prevalence of HAE is unknown, and estimates vary widely, ranging from 1 in 50,000 to 1 in 100,000 people globally. In the United States, the estimated prevalence of HAE is 5860 to 6308 cases (1.8–1.9 per 100,000), based on a 2021 study [4].

Health‐related quality of life (HRQoL) is significantly impacted by the severity and frequency of HAE attacks [5, 6, 7, 8], which interfere with patients' daily lives. These unpredictable and debilitating attacks lead to a substantial disease burden and impairments in everyday activities and work. Independent of frequency, HAE attacks can significantly impact patients' activities of daily living and HRQoL. HAE attacks adversely impact social, professional, and educational productivity and functioning [5, 9, 10, 11, 12], due to the unpredictability, pain, and severity of swelling [5, 9, 13]. Fear of attacks and fatal laryngeal attacks can lead to high rates of depression and anxiety, further reducing HRQoL [13]. A high percentage of patients with HAE experience impacts to education and career, reporting missed opportunities and hindered progression [8, 9]. The frequency and severity of attacks vary considerably. One study reported that more than two‐thirds of patients experience moderate‐to‐severe attacks, and more than three‐quarters of patients reported their most recent attack occurred within the past month [5]. Those with higher attack frequencies report greater negative impacts on daily function and diminished HRQoL [5, 13, 14]. Control of HAE attacks is correlated with HRQoL and has been identified as the most important factor influencing HRQoL [14].

Given the significant impact of HAE on patients' lives, effective treatment is crucial for improving outcomes. Current treatment strategies consist of long‐term prophylaxis, short‐term prophylaxis, and on‐demand treatment of acute attacks [15]. Early administration of on‐demand treatments is critical to accelerate symptom resolution and reduce attack severity [16] to improve patient outcomes. For patients and caregivers managing HAE, the ultimate goal is to feel symptom‐free and prevent HAE attacks [6, 16, 17]. While advancements in both acute and prophylactic treatments have been transformative [17, 18], challenges persist in optimizing patient outcomes. Specifically, delays in the early identification and treatment of acute attacks continue to contribute to poor disease control [17], despite clinical guidelines advocating for early intervention. Therefore, even with significant therapeutic advancements, a considerable disease burden for patients with HAE remains. To achieve consistent disease control and prevent long‐term complications, comprehensive and individualized treatment plans are essential.

Implementing patient‐reported outcome (PRO) measures that accurately and regularly assess attack severity may proactively identify poor disease control and facilitate tailoring treatment strategies. In the context of HAE, the Angioedema Control Test (AECT) is a 4‐item PRO measure designed to monitor disease control over a 4‐week recall period [19]. Beyond attack frequency, AECT also captures the impact of HAE attack unpredictability and frequency on patients' HRQoL. Evidence supports the construct validity and responsiveness of AECT, a subjective measure of disease control, in patients experiencing angioedema [19, 20, 21, 22, 23]. Because AECT requires minimal time and resources to administer in any healthcare setting, it is increasingly used in clinical practice (including specialized clinics and primary care offices) to assess disease burden in routine care [20, 21, 24, 25].

In addition to AECT, other PRO instruments have been developed and validated for the assessment of disease activity in patients with HAE. The Hereditary Angioedema Activity Score (HAE‐AS) is a 12‐item instrument with a recall time of “past 6 months” [26]. The Angioedema Activity Score (AAS) asks patients if they exhibited angioedema in the past 24 h, and if affirmative, patients are presented this 5‐item daily diary assessing disease activity. The AAS is generally summarized over a 7‐day or 4‐week period [27]. These tools have demonstrated utility in both clinical research and daily practice for monitoring disease activity, evaluating treatment response, and facilitating individualized patient management [16]. Incorporating a standardized assessment like AECT, HAE‐AS, or AAS into routine care can enhance the accuracy of disease monitoring and support optimal therapeutic decision‐making. This study focused on AECT due to its brevity and ease of administration [27].

Despite promising evidence of the psychometric performance of AECT, prior validation studies have primarily established its construct validity by correlating it with other patient‐reported measures [19, 20]. A critical gap remains in understanding the degree to which AECT scores correspond to an objective measure of disease control, such as investigator‐confirmed HAE attack frequency (i.e., criterion validity), specifically within the HAE population. Establishing criterion validity between AECT scores and concurrent attack frequency is an important next step toward understanding the utility of the measure in tracking patients' disease control. In addition to evaluating criterion validity with objective attack frequency, this study also examines correlations between AECT and Angioedema Quality of Life Questionnaire (AE‐QoL) scores to assess how well AECT reflects the broader subjective experience of patients, including HRQoL impacts that extend beyond symptom frequency. Therefore, this study provides the first evaluation of the criterion validity of AECT, a subjective measure of disease control, using investigator‐confirmed HAE attack rate, an objective disease control measure, in patients with HAE.

## 2 Methods

### 2.1 Data Source and Study Design

This analysis included patients with HAE enrolled in the OASIS‐HAE study: a phase 3, double‐blinded, randomized, placebo‐controlled trial (ClinicalTrials.gov Identifier: NCT05139810) designed to evaluate the safety and efficacy of donidalorsen in patients with HAE. Full details on the study design and protocol have been published elsewhere [28]. Briefly, eligible patients were aged 12 years or older at screening with confirmed type I or II HAE and experienced ≥ 2 HAE attacks as confirmed by the investigator during the screening period. Number of investigator‐confirmed HAE attacks was the primary endpoint in the OASIS‐HAE study. Patients were screened for up to 56 days, after which they were randomized in a 2:1 ratio to either a 4‐ or 8‐week dosing interval (Q4W and Q8W, respectively). Within each dosing‐interval group, patients were randomized in a 3:1 ratio to receive 24 weeks of donidalorsen 80 mg or placebo, administered subcutaneously. Treatment period visits occurred at Baseline (Week 0), and every 4 weeks after, until the end of the treatment period (Week 24).

All analyses were conducted on the full analysis population (*N* = 90), which was comprised of all randomized patients who received at least 1 injection of the study drug or placebo. For this analysis, patients were pooled across treatment arms.

### 2.2 Ethical Considerations

The OASIS‐HAE study protocol was reviewed and approved by an independent ethics committee or institutional review board at each participating site and was carried out in accordance with Good Practice Guidelines and the Declaration of Helsinki [28]. All patients provided written informed consent to participate in the study. The trial was funded by Ionis Pharmaceuticals Inc.

### 2.3 Subjective Disease Control Measure: Angioedema Control Test (AECT)

The 4‐item AECT asks patients how often they had angioedema; how much their HRQoL has been affected by angioedema; how much unpredictability of angioedema has bothered them; and how well angioedema has been controlled by their therapy. Items are scored using a 5‐point Likert scale with options ranging from “very often” or “very much” (a value of 0) to “not at all” (a value of 4) for the first 3 items, and from “not at all” (a value of 0) to “very well” (a value of 4) on the fourth item. The total score, calculated as the sum of all 4 items, ranges from 0 to 16, with total scores < 10 indicating subjective assessment of poor disease control and scores ≥ 10 indicating well‐controlled disease [20]. The highest possible score of 16 represents complete subjective disease control. AECT has previously been found to be a valid and reliable measurement tool for patients with HAE [20, 22], and was administered every 4 weeks beginning at Baseline through the 24‐week treatment period.

### 2.4 Quality of Life Measure: Angioedema Quality of Life Questionnaire (AE‐QoL)

The 17‐item AE‐QoL is a patient‐reported, symptom‐specific questionnaire that captures global impact of angioedema attacks on functioning and well‐being in patients with recurrent angioedema using a 4‐week recall period [29]. The 4 domain scores (functioning, fatigue/mood, fears/shame, and nutrition) and the total score are scaled from 0 to 100, with lower scores indicating better HRQoL. AE‐QoL has previously been found to be a valid and reliable measurement tool for patients with HAE [30, 31] and was administered at Baseline and Week 24 visits. Current analyses focus on the AE‐QoL total scores.

### 2.5 Objective Disease Control Measure: Investigator‐Confirmed HAE Attacks

To measure HAE attacks, patients were instructed to report HAE attacks within 72 h of onset and were prompted during weekly study contact visits, captured via electronic daily Angioedema Activity Score [27] questionnaire entries. Investigator‐confirmed HAE attacks were defined as events, separated by 24‐h symptom free intervals, characterized by signs or symptoms consistent with an attack in ≥ 1 pre‐specified bodily locations. Detailed information was collected about timing, location, severity, description, course, and impact of signs and symptoms, as well as treatment. The presence of symptoms was not automatically considered an HAE attack unless such a diagnosis was confirmed by the investigator. For example, investigators could have clinically determined that an event did not represent an attack if: the reported event was accompanied by symptoms that were not consistent with an HAE attack (e.g., urticaria); the reported event persisted well beyond the typical course of time for an HAE attack (e.g., greater than 7 days); or there was a likely alternative etiology for the event (e.g., viral gastroenteritis).

The monthly investigator‐confirmed HAE attack rate was calculated as the number of attacks occurring during each 28‐day interval (i.e., 4 weeks), with intervals beginning on Day 1 of the trial. The number of attacks occurring during each 4‐week period was evaluated every 4 weeks, beginning at Baseline through the 24‐week treatment period. Thus, the attack rate timeframe aligns with AECT's recall period.

### 2.6 Statistical Analysis

All analyses conducted were *post hoc*. No corrections for multiplicity of testing were applied and *p*‐values were considered nominal. No missing data imputation methods were used. Throughout the analysis, a *p*‐value of < 0.05 was considered statistically significant. Statistical analyses were performed with SAS, version 9.4 (SAS Institute Inc.; Cary, NC).

#### 2.6.1 Correlations Between AECT Score and Measures of Objective Disease Control and Health‐Related Quality of Life

##### 2.6.1.1 Correlations Between Continuous AECT Scores and HAE Attack Rates

Associations between continuous AECT scores and HAE attacks over the previous 4 weeks were evaluated at Baseline, Week 12, and at Week 24 visits. Spearman rank‐order correlation coefficients (*ρ*) were used. Given that higher HAE attack rates were expected among patients with subjective assessment of poor disease control, it was hypothesized a priori that negative correlations would be observed between AECT scores and HAE attack rates, as better disease control is indicated by higher AECT scores and lower HAE attack rates. To visualize the cross‐sectional association between the continuous AECT scores and HAE attack rates, bubble plots for Baseline, Week 12, and Week 24 were created. Larger bubbles indicated a greater number of patients with a specific combination of AECT score and HAE attack rate.

##### 2.6.1.2 Correlations Between Change in AECT Score and Change in HAE Attack Rate

In addition, the associations between change in AECT score and change in HAE attack rate were examined using Spearman correlation coefficients. Changes were calculated from Baseline to Week 12 and from Baseline to Week 24 visits. It was hypothesized a priori that negative correlations would be observed between changes in continuous AECT scores and change in HAE attack rates over time. Bubble plots were created to visualize the results with larger bubbles indicating a greater number of patients with a specific combination of change in AECT score and change in HAE attack rate.

##### 2.6.1.3 Correlations Between AECT Scores and AE‐QoL Total Scores

Pearson correlations (*r*) assessed the extent to which AE‐QoL total scores were associated with AECT scores at Baseline and Week 24, as well as the associations between change in AECT score and change in AE‐QoL total score. Given that higher AECT scores indicate better disease control and lower AE‐QoL scores indicate better HRQoL, it was hypothesized a priori that scores would be moderately negatively correlated.

#### 2.6.2 Subjective Disease Control

##### 2.6.2.1 Poor (AECT Score < 10) Versus Well‐Controlled Disease (AECT Score ≥ 10)

To compare HAE attack rates of patients with subjective assessment of poor disease control (i.e., AECT score < 10) to patients assessing their disease to be well‐controlled (i.e., AECT score ≥ 10) at Baseline, Week 12, and Week 24, descriptive statistics, independent‐sample *t*‐tests, and Cohen's *d* effect size (ES) estimates for standardized mean differences were used. Throughout the analysis, Cohen's *d* ES were interpreted according to Cohen's published guidelines (i.e., *d* < 0.2, negligible effect; *d* ≥ 0.2 to 0.5, small effect; *d* ≥ 0.5 to 0.8, medium effect; *d* ≥ 0.8, large effect) [32].

To visualize the distribution of the groups, box and whisker plots of the rate of HAE attacks among patients with AECT scores < 10 and ≥ 10 were generated for Baseline, Week 12, and Week 24 visits.

##### 2.6.2.2 Less Than Complete (AECT Score < 16) Versus Complete (AECT Score of 16) Disease Control

In addition, HAE attack rates for patients with subjective assessment of less than complete disease control (i.e., AECT score < 16) and patients who assessed their disease to be completely controlled (i.e., AECT score of 16) were compared at Week 12 and Week 24 using descriptive statistics, independent‐samples *t*‐tests, and Cohen's *d* ES. Box and whisker plots for the rate of HAE attacks over the previous 4 weeks among patients with subjective assessment of less than complete and complete disease control were created for Baseline, Week 12, and Week 24 visits, to visualize the distribution of the groups.

#### 2.6.3 Objective Disease Control

##### 2.6.3.1 No HAE Attacks Versus at Least One HAE Attack

To compare AECT scores between patients with objectively complete disease control (i.e., HAE attack rate of 0) and not completely controlled disease (i.e., HAE attack rates > 0), at Week 12 and Week 24, descriptive statistics, independent samples *t*‐tests, and Cohen's *d* ES were used. Analyses were performed only if the sample size for the subgroup of patients with HAE attack rates of 0 at the visit was ≥ 5.

## 3 Results

The analytic sample consisted of 89 individuals at Baseline; at the end of treatment (Week 24), the cohort consisted of 85 individuals. All individuals were treated either Q4W or Q8W with donidalorsen 80 mg or placebo for 24 weeks.

### 3.1 Correlations Between AECT Scores and Measures of Objective Disease Control and Health‐Related Quality of Life

#### 3.1.1 Correlations Between Continuous AECT Scores and HAE Attack Rates

**FIGURE 1:**
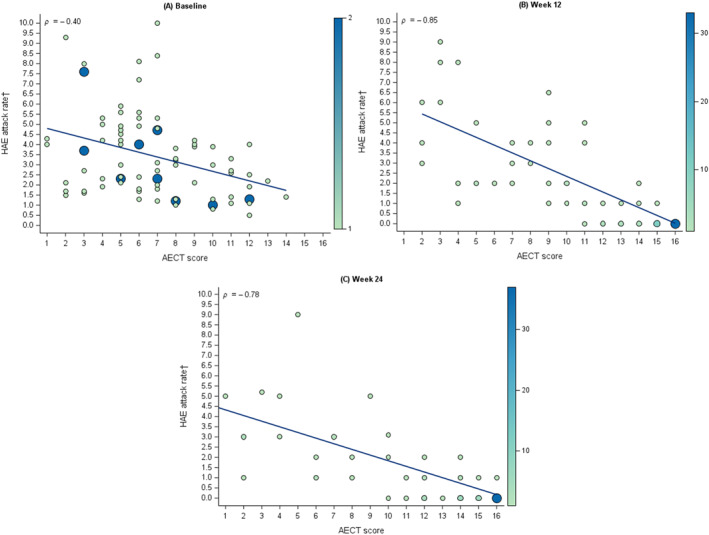
Relationship between AECT score and HAE attack rate^†^ in patients with HAE, at (A) Baseline, (B) Week 12, and (C) Week 24. The size of each bubble corresponds to the count of participants with that distinct AECT score and HAE attack rate combination. The solid trend lines depict the general relationship between AECT scores and HAE attack rates, supported by Spearman's rank correlation coefficient (*ρ*). AECT, Angioedema Control Test; HAE, hereditary angioedema. †HAE attack rate is defined as the number of investigator‐confirmed HAE attacks per 4 weeks. ^†^HAE attack rate is defined as the number of investigator‐confirmed HAE attacks per 4 weeks.

Moderate‐to‐large negative Spearman correlations were observed between AECT scores and HAE attack rate at Baseline (*ρ* = −0.40), Week 12 (*ρ* = −0.85), and Week 24 (*ρ* = −0.78), in accordance with prespecified hypotheses. These correlations indicate strong associations between continuous AECT scores and HAE attack rates at post‐Baseline visits (Figure 1).

### 3.2 Correlations Between Change in AECT Score and Change in HAE Attack Rates

**FIGURE 2:**
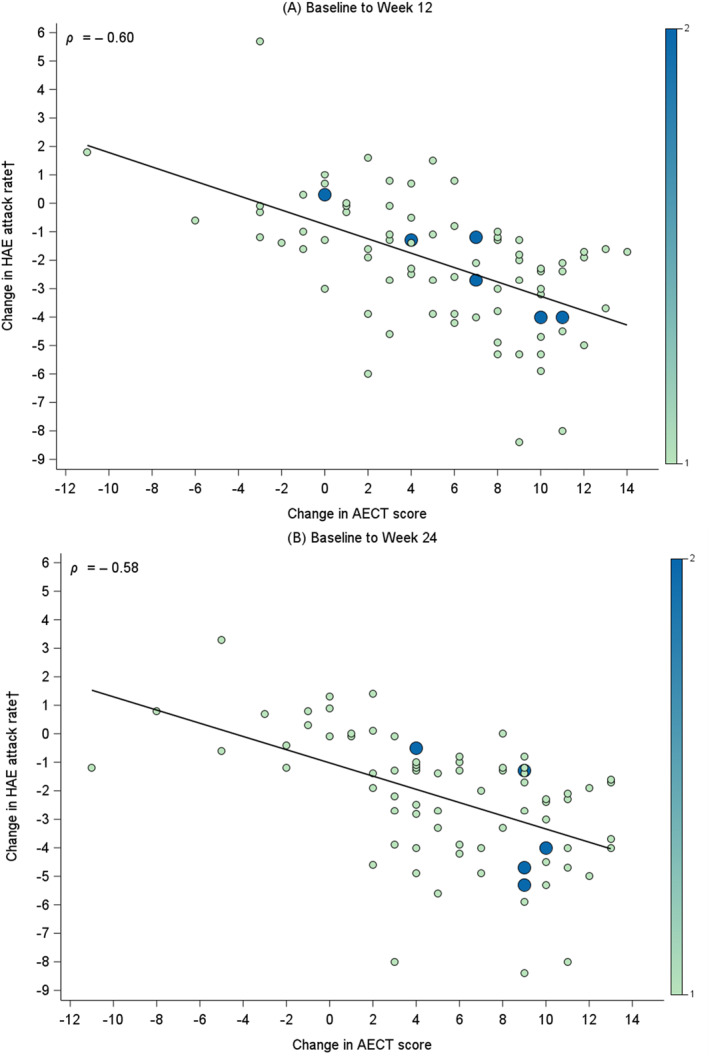
Relationship between change in AECT score and change in HAE attack rate^†^ in patients with HAE, from: (A) Baseline to Week 12 and (B) Baseline to Week 24. The size of each bubble corresponds to the count of participants with that distinct AECT score and HAE attack rate combination. The solid trend lines depict the general relationship between AECT scores and HAE attack rates, supported by Spearman's rank correlation coefficient (ρ). AECT, Angioedema Control Test; HAE, hereditary angioedema. †HAE attack rate is defined as the number of investigator‐confirmed HAE attacks per 4 weeks.

In accordance with prespecified hypotheses, a large negative Spearman correlation was observed between change in AECT scores and change in HAE attack rate from Baseline to Week 12 (*ρ* = −0.60) and from Baseline to Week 24 (*ρ* = −0.58). These correlations indicate strong associations between changes in both AECT scores and HAE attack rates across study visits (Figure 2).

#### 3.2.1 Correlations Between AECT Scores and AE‐QoL Total Scores

As hypothesized, AECT scores had moderate, negative correlations with AE‐QoL total scores at both Baseline (*r* = −0.59) and Week 24 (*r* = −0.77). Change in AECT score also moderately negatively correlated with change in AE‐QoL total score from Baseline to Week 24 (*r* = −0.67).

### 3.3 Subjective Disease Control

Table 1 illustrates the distribution of HAE attack rates within each subgroup at each time point.

**TABLE 1:** Differences in mean HAE attack rates^a^ between patients with varying levels of subjective disease control as measured by AECT, across study visits.

(A) Patients with subjective assessment of poor versus well‐controlled disease	Patient assessment of poor disease control (AECT score < 10)			Patient assessment of well‐controlled disease (AECT score ≥ 10)			*p*‐value^b^	ES, Cohen's *d*
	*n*	Mean HAE attack rate	SD	*n*	Mean HAE attack rate	SD		
Baseline HAE attack rate	69	3.74	2.15	20	1.92	1.04	**<** **0.001**	−1.08
Week 12 HAE attack rate	23	4.02	2.32	66	0.33	0.90	**<** **0.001**	−2.10
Week 24 HAE attack rate	18	3.29	2.03	67	0.24	0.61	**<** **0.001**	−2.03

(B) Patients with subjective assessment of less than complete versus complete disease control	Patient assessment of less than complete disease control (AECT score < 16)			Patient assessment of complete disease control (AECT score of 16)			*p*‐value^b^	ES, Cohen's *d*
	*n*	Mean HAE attack rate	SD	*n*	Mean HAE attack rate	SD		
Baseline HAE attack rate	89	3.33	2.10	0	—	—	—	—
Week 12 HAE attack rate	56	2.04	2.40	33	0.00	0.00	**<** **0.001**	−1.21
Week 24 HAE attack rate	47	1.58	1.95	38	0.03	0.16	**<** **0.001**	−1.12

*Note:* Bolded values indicate statistical significance at *p*‐value < 0.05.

Abbreviations: AECT, Angioedema Control Test; ES, effect sizes; HAE, hereditary angioedema; SD, standard deviation.

^a^ HAE attack rate is defined as the number of investigator‐confirmed HAE attacks per 4 weeks.

^b^ *p*‐value is based on an independent‐samples *t*‐test comparing the mean HAE attack rates between the two disease control subgroups (e.g., poor‐vs. well‐controlled).

#### 3.3.1 Subjective Assessment of Poor (AECT Score < 10) Versus Well‐Controlled Disease (AECT Score ≥ 10)

In accordance with a priori hypotheses, HAE attack rates were significantly and substantially larger for patients with subjective assessment of poor disease control than for patients assessing their disease to be well‐controlled (all *p* < 0.001; Table 1[a]). Mean HAE attack rates were higher among patients with assessment of poor disease control compared with the attack rates among patients who assessed their disease to be well‐controlled. ES for pairwise differences were very large for HAE attack rates at all 3 visits (range: ES = −1.08 to −2.10; Table 1[A]).

#### 3.3.2 Subjective Assessment of Less Than Complete (AECT Score < 16) Versus Complete (AECT Score of 16) Disease Control

In accordance with a priori hypotheses, HAE attack rates were significantly and substantially larger for patients with subjective assessment of less than complete disease control than for patients who assessed their disease to be completely controlled (all *p* < 0.001; range ES = −1.12 to −1.21; Table 1[B]).

**FIGURE 3:**
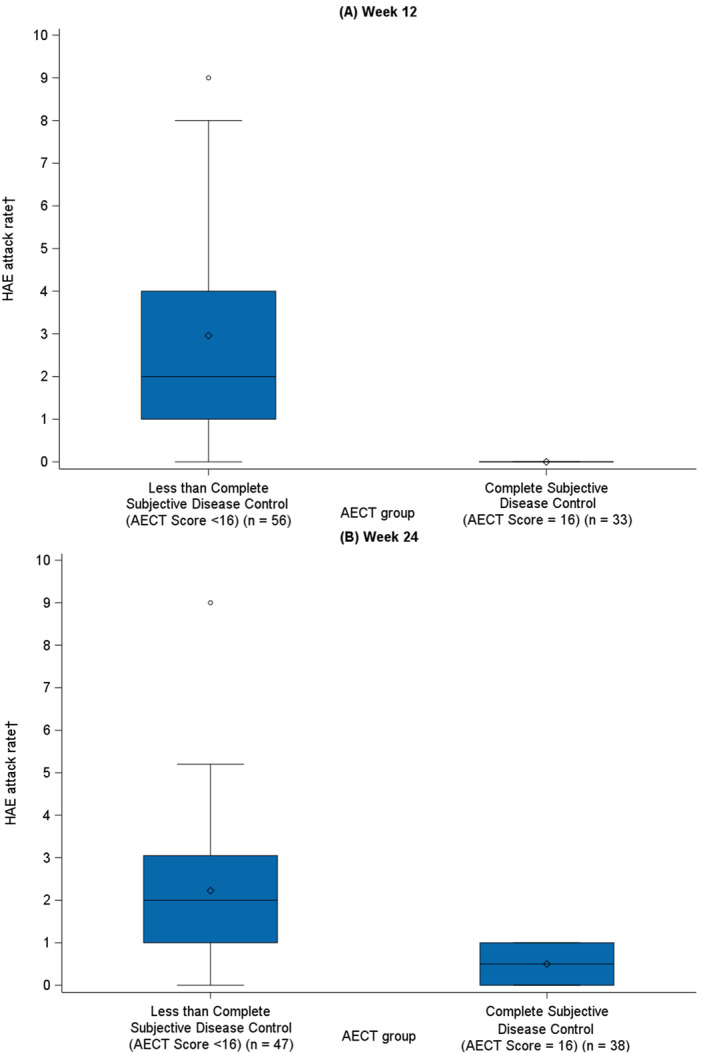
Mean HAE attack rates^†^ between patients with less than complete (AECT Score < 16) and complete (AECT Score of 16) subjective disease control at (A) Week 12 and (B) Week 24. The box plots illustrate the distribution of HAE attack rates for patients with less than complete versus complete subjective disease control, defined by AECT score. The central horizontal line within each box represents the median score, the individual point represents the mean score, and the box itself encompasses the IQR (25th to 75th percentiles). Whiskers extend to the most extreme data points within 1.5 times the IQR, and individual points represent outliers falling outside this range. The boxes for the complete disease control group depict a lower median attack rate and smaller IQR compared to the less than complete disease control group. AECT, Angioedema Control Test; HAE, hereditary angioedema; IQR, interquartile range. †HAE attack rate is defined as the number of investigator‐confirmed HAE attacks per 4 weeks.

Figure 3 illustrates the distribution of HAE attack rates within each subgroup at each time point. Of the 38 patients with completely controlled disease at Week 24, 37 had an attack rate of 0, with the remaining patient having 1 attack (Figure 3).

### 3.4 Objective Disease Control

#### 3.4.1 No HAE Attacks Versus at Least One HAE Attack

Descriptive summary statistics (mean and standard deviation [SD]) and tests of magnitudes of differences for AECT scores in patients with no HAE attacks (i.e., HAE attack rate of 0) versus patients with at least one HAE attack are shown in Table 2. In accordance with a priori hypotheses, AECT scores were significantly and substantially larger for patients with no HAE attacks than for patients with at least one HAE attack (all *p* < 0.001; range ES = 2.14 to 2.65; Table 2).

**TABLE 2:** Differences in mean AECT scores between patients with at least one HAE attack (HAE attack rates^a^ > 0) and patients with no HAE attacks (HAE attack rate of 0) across study visits.

	HAE attack rates > 0			HAE attack rate of 0			*p*‐value^b^	ES, Cohen's *d*
	*n*	Mean AECT score	SD	*n*	Mean AECT score	SD		
Baseline AECT scores	87	6.86	3.09	0	—	—	—	—
Week 12 AECT scores	35	7.77	3.75	54	15.20	1.29	**<** **0.001**	2.65
Week 24 AECT scores	29	7.66	4.71	56	15.14	1.49	**<** **0.001**	2.14

*Note:* Bolded values indicate statistical significance at *p*‐value < 0.05.

Abbreviations: AECT, Angioedema Control Test; ES, effect sizes; HAE, hereditary angioedema; SD, standard deviation.

^a^ HAE attack rate is defined as the number of investigator‐confirmed HAE attacks per 4 weeks.

^b^ *p*‐value is based on independent‐samples *t*‐test comparing the mean AECT scores between patients with and without HAE attacks.

The distribution of AECT scores among patients with HAE attack rates of 0 over the previous 4 weeks (i.e., no HAE attacks) were informative. The majority of patients with 0 HAE attacks reported an AECT score of 16, with no patients reporting scores less than 10, both at Week 12 (mean: 15.20; SD: 0.18; range: 11–16) and at Week 24 (mean: 15.14; SD: 0.20; range: 10–16).

## 4 Discussion

Study findings demonstrate the criterion validity of AECT in a sample of patients with HAE. Moderate‐to‐strong correlations were observed between AECT scores and HAE attack rates, both at specific time points and for change in both measures over the study duration. Patients who assessed their disease as well‐controlled on AECT had substantially lower HAE attack rates, and patients who reported completely controlled disease had an HAE attack rate of nearly 0. Finally, the group of patients with no reported HAE attacks had mean AECT scores > 15. Additionally, correlations with AE‐QoL indicate that AECT captures subjective aspects of a patient's experience, beyond attack frequency, providing a more holistic view of disease control.

In conditions characterized by recurrent attacks and fluctuating disease activity, such as HAE, the ability to assess disease control is imperative. To adequately manage treatment and maintain disease control for their patients, physicians need to understand the severity and frequency of attacks, current disease activity, how well the disease is controlled, and the impact of the disease on patients' daily lives and HRQoL [33]. A short, standardized, self‐reported questionnaire to assess disease control in patients with HAE may provide a useful measure of disease control that could improve treatment planning and decision making in clinical practice. Early identification of poor disease control and prompt treatment results in less severe attacks both in intensity and duration [12]. The current study adds to this evidence base by demonstrating criterion validity between AECT and investigator‐confirmed HAE attack rates in this population and suggests that AECT may be a pragmatic and useful tool for measuring disease control in clinical settings. The present findings support the use of AECT as a pragmatic tool for monitoring disease activity in HAE. Other PRO instruments, including the HAE‐AS and AAS, may also be useful in clinical practice, and future research could compare the ease of administration, performance, and utility of these instruments in diverse patient populations.

Previously, AECT has been used in clinical research, but not to evaluate the efficacy of new HAE treatments. Its brevity and accuracy indicate that it could be an important tool in health management for patients with HAE and their physicians [20]. AECT can help physicians make informed decisions about treatment strategies, such as whether a treatment regimen is working effectively or may need adjustments. AECT can also help provide a quantifiable way to track and assess treatment effectiveness and clinically important changes in disease control over time [21]. So far, its use in real‐world clinical settings has been limited, but it may be a valuable tool for monitoring patient health [25]. The substantial correlations between AECT and AE‐QoL, which measures the global impact of angioedema attacks on functioning and well‐being [29], including data presented here and in previous research, further substantiates AECT's value [20, 21, 22, 34]. This indicates AECT's potential as a disease control monitoring tool that effectively predicts attack frequency and captures the subjective patient experience of disease control in HAE. AECT can provide a structured framework in clinical settings for patients to communicate their experience and symptoms, facilitating more effective communication and collaboration with their physicians, improving their disease management via improved disease control monitoring, and ultimately improving their HRQoL.

This study has several limitations, and its findings should be interpreted with caution. A primary limitation is that data were sourced from a clinical trial, which does not fully mirror a real‐world setting. Clinical trial participants are carefully selected based on strict eligibility criteria and may not represent the broader, more heterogeneous population of patients with HAE who often have comorbidities. Therefore, the findings may not be generalizable to all patients with HAE in routine clinical practice. Additional evidence collected in patients with HAE in a real‐world study or clinical setting is necessary to contextualize and confirm the findings from this study. Additionally, analyses performed were exploratory and post hoc with no control for multiplicity; this increases the risk of Type I errors. Finally, patient responses to some items in AECT may have been impacted by their interpretation or understanding, as well as by their participation in the clinical trial. The first item in AECT (“In the last 4 weeks, how often have you had angioedema?”) may be confusing for patients with HAE, who may confound the symptom(s) of angioedema, which vary, with the condition of angioedema, which is invariable. As a result, some patients with HAE may respond thinking they always have the condition, rather than answering about symptoms. More research is needed to elucidate the patient perspective of this language. Additionally, the fourth item in AECT (“In the last 4 weeks, how well has your angioedema been controlled by your therapy?”) may be more easily answered when patients are using long‐term prophylaxis. As such, the trial population may have had more focus on completing this item than the average patient in a clinic, due to their trial treatment and participation. This study did not directly compare AECT with other disease activity assessment tools such as the HAE‐AS or AAS, which may be considered in future research. Given these limitations, this study's findings should be considered preliminary and exploratory. Further research with appropriate statistical controls and a larger, more representative sample is needed to confirm and generalize the results.

In conclusion, this study provides novel evidence for the criterion validity of AECT by demonstrating a significant correlation between AECT scores and an objective measure of HAE attack frequency, supporting the potential of AECT as a valuable tool for monitoring disease control in patients with HAE. AECT was associated with frequency of HAE attacks in this clinical trial sample of patients with HAE. Integrating this short, standardized survey into clinical practice could facilitate more effective communication between patients and physicians, leading to improved treatment outcomes, and enhanced HRQoL.

## Author Contributions

**Aaron Yarlas:** conceptualization, methodology, writing – review and editing, funding acquisition, supervision. **Alexandra J. Feld:** conceptualization, methodology, visualization, writing – original draft, writing – review and editing, formal analysis. **Jakob B. Bjorner:** conceptualization, methodology, writing – review and editing, formal analysis, supervision. **Cary Thurm:** conceptualization, writing – review and editing, software, formal analysis. **Laura Bordone:** conceptualization, writing – review and editing. **Kenneth B. Newman:** conceptualization, writing – review and editing. **Sabrina Treadwell:** conceptualization, writing – review and editing. **Danny M. Cohn:** conceptualization, writing – review and editing, supervision. All authors reviewed and approved the final manuscript.

## Funding

This study was supported by Ionis Pharmaceuticals Inc.

## Conflicts of Interest

A.Y., L.B., K.B.N. and S.T. are employees of and hold shares and/or options in Ionis Pharmaceuticals Inc., which funded this research. A.J.F., J.B.B. and C.T. are employees of IQVIA, which received funding from Ionis to conduct this research. D.M.C. reports speaking fees from CSL Behring, Intellia, Ionis, Pharvaris, and Takeda; reports consultancy fees from Astria, BioCryst, CSL Behring, Ionis, KalVista, Pharming, Pharvaris, and Takeda; and reports research support from Ionis, KalVista, Pharvaris, and Takeda.

## Data Availability

The authors have nothing to report.
